# Short clones or long clones? A simulation study on the use of paired reads in metagenomics

**DOI:** 10.1186/1471-2105-11-S1-S12

**Published:** 2010-01-18

**Authors:** Suparna Mitra, Max Schubach, Daniel H Huson

**Affiliations:** 1 Center for Bioinformatics ZBIT, Tübingen University, Sand 14, 72076 Tübingen, Germany

## Abstract

**Background:**

Metagenomics is the study of environmental samples using sequencing. Rapid advances in sequencing technology are fueling a vast increase in the number and scope of metagenomics projects. Most metagenome sequencing projects so far have been based on Sanger or Roche-454 sequencing, as only these technologies provide long enough reads, while Illumina sequencing has not been considered suitable for metagenomic studies due to a short read length of only 35 bp. However, now that reads of length 75 bp can be sequenced in pairs, Illumina sequencing has become a viable option for metagenome studies.

**Results:**

This paper addresses the problem of taxonomical analysis of paired reads. We describe a new feature of our metagenome analysis software MEGAN that allows one to process sequencing reads in pairs and makes assignments of such reads based on the combined bit scores of their matches to reference sequences. Using this new software in a simulation study, we investigate the use of Illumina paired-sequencing in taxonomical analysis and compare the performance of single reads, short clones and long clones. In addition, we also compare against simulated Roche-454 sequencing runs.

**Conclusion:**

This work shows that paired reads perform better than single reads, as expected, but also, perhaps slightly less obviously, that long clones allow more specific assignments than short ones. A new version of the program MEGAN that explicitly takes paired reads into account is available from our website.

## Background

Metagenomics is the study of environmental samples using sequencing [1], focusing on microbes that cannot be studied in pure culture. Rapid advances in sequencing technology are currently fueling a vast increase in the number and scope of metagenomics projects [2].

The analysis of metagenomic datasets is an immense conceptual and computational challenge, and there is a great need for new bioinformatics tools and methods. However, this has so far, largely escaped the notice of the bioinformatics community. Indeed, the term "Metagenomics" does not appear in the main call for papers for any of this year's international bioinformatics conferences, including APBC, ISMB, RECOMB and WABI.

The two first main computational problems in metagenomics are to estimate the taxonomical content and the functional content of a given dataset. A further task is to compare the contents of different metagenomic datasets. The difficulty of these challenges stems from the huge amounts of data to be processed, the poor sampling of reference sequences, the lack of adequate models for data acquisition and the demands of statistical analysis.

A number of facilities provide dedicated computational resources or services for metagenomics, including SEED [3], IMG/M [4] and CAMERA [5]. A number of publications have described new computational approaches (see [6] for an overview). However, many of these are of limited practical use because the authors make little attempt to provide robust and user-friendly implementations of their methods.

In [7], we published the first available stand-alone metagenomic analysis tool, called MEGAN. The program now has over 1000 registered users and has been used in a number of publications, including [8-13]. To analyze a metagenomic dataset using MEGAN, the dataset is first compared against a reference database. For example, non-specific DNA samples can be compared against the NCBI-nr database [14] using BLASTX [15], datasets targeting viruses can be blasted against the NCBI viral genome database, and ribosomal RNAs can be compared against a dedicated RNA database [8]. The program uses an "LCA-gene content" algorithm to perform taxonomical analysis, placing reads on nodes at different levels of the NCBI-taxonomy, in a way that reflects the presence or absence of homologous genes in different species. The program also provides a comparative view of multiple datasets [16]. Moreover, the next release will provide a functional analysis utilizing the Gene Ontology [17].

Many important metagenome projects are based on Sanger sequencing, for example [10,18,19]. The main advantage of Sanger sequencing is that the reads can be up to 1,000 bp in length. Such long reads are desirable for a number of reasons. First, longer reads usually give rise to longer and better matches to reference sequences, and so such reads can be assigned to specific taxa with higher confidence. Second, reads of this length can contain whole open reading frames and thus are very useful for finding new genes. Finally, the problem of assembling the most abundant species in a metagenome, when desired, is easier for longer reads. The main draw-back of Sanger sequencing is the high price per base pair.

The first of the so-called "next generation" sequencing technologies, Roche-454 sequencing [20], has become more and more popular as an alternative to Sanger sequencing. Originally producing reads of about 100 bp in length, the attainable read length then grew to 250 bp, and now a read length of over 400 bp is possible, for a much lower price per base pair than Sanger sequencing.

Until quite recently, the second next-generation sequencing technique to become commercially available, Illumina sequencing [21], was not considered suitable for metagenomic studies because of its short read length in the range of 35 bp. Recent improvements support a read length of 75 bp, and such reads can now be collected in a paired-read protocol. Illumina sequencing has become an even cheaper option for metagenome sequencing.

The paired reads are sequenced from the two ends of individual fragments, which we will call *clones*. Using two different protocols, Illumina sequencing supports the paired-end sequencing of two different lengths of clones: *short clone *libraries of an average length of 200 bp, say, and *long clone *libraries, of an average length of 2,000 bp. For more details, see the supplementary material of [22].

In this paper, we report on a simulation study that we have undertaken to compare the performance of the LCA-gene content algorithm for different types of sequencing technologies. Our main goal is to determine whether it is better to apply paired-end sequencing to short clones or to long clones when performing a taxonomical analysis of a metagenomic dataset. Also, we compare the use of Illumina paired reads to the use of Illumina single reads and Roche-454 single reads. Our performance study is focused on MEGAN, as other available tools do not explicitly make use of paired-end reads.

Our main results are that homology-based taxonomical analysis algorithms such as the LCA-gene content approach implemented in MEGAN can produce more specific taxonomical assignments of reads, when they are modified to combine the matches from explicitly paired reads, as one would expect. Moreover, perhaps less obviously, using long clones, rather than short ones, show the best increase of specificity.

## Results and discussion

### Taxonomical analysis by homology and gene content

The diversity of the microbial world is believed to be huge. However, only about 6,000 microbial species have been named [23] and many of these are represented by only a few genes, at most, in public sequence databases. Moreover, current databases are biased toward organisms of specific interest and were not explicitly populated to represent a wide sampling of biodiversity. For this reason, taxonomical analysis currently cannot be based on high similarity sequence matching, but rather depends on the detection of homologies using quite sensitive methods.

One approach is to use *phylogenetic markers *to distinguish between different species in a sample. The most widely used marker gene is the SSU rRNA gene; others include RecA, EF-Tu, EF-G, HSP70 and RNA polymerase B (RpoB) [18]. Advantages of this approach are that such genes have been studied in detail and for some there are high quality phylogenies available that can be used as a reference to place reads from metagenomic dataset. However, this approach is not unproblematic: On the one hand, the use of "universal" primers to target specific genes suffers from the problem that such primers are not truly universal and so only a portion of the true diversity is captured. On the other hand, while the use of a random shotgun approach can overcome this problem, less than 1% of the reads in a random shotgun dataset will correspond to commonly used phylogenetic marker genes [24], which seems very wasteful, as 99% percent of the reads will remain unused (and unclassified).

Moreover, the goal of taxonomical analysis is not only to provide an estimation of the types of organisms present in a sample, but also to corral the sequence reads by taxonomical identity to facilitate further analysis, for example to study the GC content or to attempt the assembly of particular genomes.

Our approach is to compare reads against the NCBI-nr database (or some other appropriate database) to find homologous sequences, thus making use of the fact that homologies are easier to detect on the protein level. For the above-mentioned reason that current databases provide only a poor coverage of the true diversity of organisms, we treat all sequence matches of high significance as equally valid indications that the given read represents a gene that is present in the corresponding organism. In more detail, we place each read on the lowest common ancestor (in the NCBI taxonomy) of all the organisms that are known to contain the gene present in the read. So, in essence, the placement of a read is governed by the gene content of the available reference genomes and thus we will refer to our method as the *LCA-gene content *approach.

An attractive feature of this "LCA-gene content" approach is that it is inherently conservative and is more prone to err toward non-informative assignments of reads (to high-level nodes in the taxonomy) than toward false-positive assignments (placing reads from one species onto the node of another species). In particular, genes that are susceptible to horizontal gene transfer will not be assigned to any of the participating species, as long as more than one is hit in the reference database.

### Short clones or long clones?

Most metagenome projects have used either Sanger sequencing or Roche-454 sequencing. However, the *Illumina Genome Analyzer *now produces reads of length 75 bp in pairs. As mentioned above, these pairs can either come from short clones of, say, 200 bp length or from long clones, that can span 2 kb, say.

Thus, Illumina sequencing using paired-end reads now appears to be a viable alternative to Roche-454 or Sanger sequencing for taxonomical analysis in metagenomics. The question arises how the two different paired-read protocols compare with each other, and also with Illumina and Roche-454 single read sequencing. (Because the Roche-454 pair-end protocol requires an additional cloning step, it is not used in metagenomics).

As mentioned above, the LCA-gene-content approach implemented in MEGAN suffers primary from a lack of resolution. A read that has a highly significant match to a sequence in the NCBI-nr database will often match similar sequences from other organisms, as well, and thus may be placed on a higher-level taxon.

**Figure 1:**
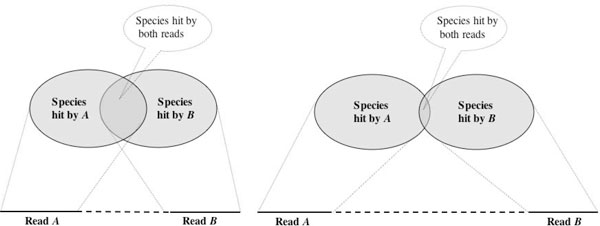
**Short clones vs long clones**. We assume that the intersection of the species hit by two reads *A *and *B *will be larger for pair reads obtained from short clones than for ones obtained from long clones. If this is the case, then use the of long clones in metagenome projects should lead to a more specific assignment of reads.

Assume that we have a set of reads collected using a paired-read protocol. If we process two reads from the same clone simultaneously, then the distance between the two reads in the source genome (i.e. the length of the clone from which they were sequenced) will affect the performance of the LCA-gene content algorithm: If the two reads are close together, as in the case of short clones, then it is more likely that the two reads will come from the same gene and thus will display the same pattern of hits among species. If, on the other hand, the two reads lie much further apart in the source genome, as in the case of long clones, then it is more likely that the reads will come from two different genes, and these might show quite different patterns of conservation among species (see Figure 1).

Indeed, in the simulations reported below, we observed that whenever the two reads of a short clone (≈ 200 bp) matched the same taxon, then this is due to matches to the same gene in over 80% of the cases, whereas for long clones (≈ 1,900 bp) this is true for just under 12%.

Thus, if we modify the LCA-gene content algorithm to place more weight on those species that are hit by both reads, then it should be the case that using long clones will give rise to more specific taxonomical assignments that when using short clones, without increasing the number of false-positive assignments. Moreover, it should, of course, be the case that processing both reads of a pair together will provide better results than processing each read in isolation.

### Processing paired reads in MEGAN

Reads from metagenomic datasets are usually processed in isolation (unless an assembly is attempted). MEGAN has a pre-computational step where each read is blasted against a reference database such as NCBI-nr. MEGAN filters the matches obtained for a read by bit score. First, only matches that exceed a minimal bit score of 35, say, are kept (this is called the *min score *filter). Second, the hits are filtered further so that only those that attain a score that is within 10% (say) of the best score seen for the given read are kept (the *top percent *filter). For each hit that passes these two filters, MEGAN determines the corresponding species and then assigns the read to the LCA of the species of all hits, as outlined above. A third filter, called the *min support *filter is then applied which removes all taxa from the reported result that were not hit by a specific number of reads.

To accommodate paired reads, we have implemented a new *paired-reads mode *in MEGAN. After importing all reads, MEGAN processes each pair of reads in turn. In more detail, matches to the same organism from the two different reads are treated as one match. To give one of these paired matches more weight, we propose to combine the bit scores *s*_1 _and *s*_2 _from the two reads using the following calculation:

with *r *= 2, *k *= 0.041 (database parameter reported by BLAST), gap size *g *= 50, effective length of the query *m' *=  (For BLASTX *m' *= ), query length *m*, effective HSP *h*, effective length of the subject *n' *=  and subject length *n*. For more details on these parameters, see [25].

The number of organisms that are hit by both reads of a pair will often be smaller than the number of different organisms that are hit by either of the reads on their own. The modified bit score of two combined hits will often be more than 10% higher than the score of uncombined hits and so, in many cases, only the combined matches will pass the 10% filter. In consequence, the resulting LCA placement should be more specific.

## Conclusion

In this paper, we have investigated the question whether the taxonomical analysis of metagenomic datasets can be performed by Illumina paired reads, and, if so, whether short clones or long clones should be used. Our simulation study suggests that Illumina paired reads are well suited for this task and that long clones are more specific, even compared to much longer Roche-454 reads (of length 250 bp), when using the LCA-gene content algorithm. We have argued that this is due to the fact that the placement of reads from long clones are based on the gene-content pattern of two different genes, rather than just one. This is a general observation that will probably affect other analysis methods that consider paired reads, as well.

Because Illumina sequencing is much cheaper than Roche-454 sequencing, it is clear that future metagenomics projects will use Illumina sequencing, as well as Sanger and Roche-454 sequencing. To support the analysis of such datasets, we have modified our program MEGAN explicitly to make use of paired reads in taxonomical analysis.

As the size and number of metagenomic datasets continue to grow, a major challenge is to significantly speed-up the sequence comparison step, while maintaining the sensitivity of BLAST. Unfortunately, the many new fast read-mapping tools that have been developed for mapping short reads, in the context of resequencing, say, are not immediately applicable as they do not map DNA to proteins.

## Methods

### Simulation of metagenomes and sequencing

We used the MetaSim simulator [26] to simulate the sequencing of three different synthetic metagenomes of different complexities, using Roche-454 sequencing, Illumina paired-end sequencing of short clones and Illumina pair-end sequencing of long clones, as described in more detail below. For a fair comparison, the ratio of the total number of base pairs simulated for the Roche-454 and Illumina technologies was 1:10, based on the assumption that the price ratio between Roche-454 sequencing and Illumina paired-end sequencing is roughly of that order.

The three synthetic metagenomes were put together using whole-genome prokaryotic sequences downloaded from the NCBI website (April 2009), in accordance with the three profiles described in [27]. In more detail, the three metagenomes are:

• A low complexity (LC) metagenome, consisting of 104 species and featuring one highly abundant species *Rhodopseudomonas palustris*;

• A medium complexity (MC) metagenome, consisting of the same 104 species including 6 highly abundant species: *Xylella fastidiosa Dixon, Rhodopseudomonas palustris BisB5, Bradyrhizobium sp. BTAi1, Xylella fastidiosa Ann-1, Rhodopseudomonas palustris BisB18 *and *Rhodospirillum rubrum ATCC 11170*;

• A high complexity (HC) metagenome, consisting of the same 104 species, all at similar levels of abundance.

(Nine taxa mentioned in [27] were not found in the NCBI database and thus were omitted from our analysis. Their taxon ids are: 155920, 155919, 165597, 332415, 322710, 286604, 321955, 333146 and 333849.)

We simulated Roche-454 reads for each of these three datasets with MetaSim using a setting of 98 flow cycles to obtain reads that are ≈ 250 bp in length. MetaSim models the basic base-calling procedure of Roche-454 sequencing. However, additional corrective post-processing is not simulated and so the errors reported here may be higher than what one would encounter in practice. For each dataset, we produced 6,000 (non-paired) reads (see Table 1).

**Table 1:** Roche-454 reads statistics. Summary of the Roche-454 reads generated by MetaSim for each of the three synthetic metagenome datasets LC, MC and HC.

	LC-454	MC-454	HC-454
Simulated reads	6,000	6,000	6,000
Simulated base pairs	1,548,902 bp	1,541,252 bp	1,573,651 bp
Average read length	258.15 bp	256.88 bp	262.28 bp
Insertions	35,796 (2.3%)	35,425 (2.3%)	36,013 (2.3%)
Deletions	8,911 (0.5%)	8,839 (0.5%)	9,208 (0.5%)
Substitutions	0	0	0

For each of the three synthetic metagenomes, we produced two different set of Illumina reads with the goal of simulating the sequencing of both short clone and long clone libraries. The current release of the MetaSim software provides an error profile for Illumina reads of length 36 bp. To obtain an error profile for longer reads of length 75 bp, we applied a non-linear regression (*f*(*x*) = *a*·*e*^*b*·*x *^+ *c*) to produce the best fitted error model (*a *= 3.957*e *- 4, *b *= 1.319*e *-1 and *c *= 5.362*e *- 3).

For the short clone library (S), we set MetaSim to generate clones according to a normal distribution with *μ *= 200 bp and *σ *= 20 bp (see Table 2). For the long clone library (L), we set MetaSim to generate clones according to a normal distribution with *μ *= 1,900 bp and *σ *= 300 bp (see Table 3).

**Table 2:** Illumina short-clone reads statistics. Summary of the Illumina short-clone reads generated by MetaSim for each of the three synthetic metagenome datasets LC, MC and HC.

	LC-ilm-S	MC-ilm-S	HC-ilm-S
Simulated reads	200,000	200,000	200,000
Read length	75 bp	75 bp	75 bp
Clone length	200 bp	200 bp	200 bp
Simulated base pairs	15,000,000	15,000,000	15,000,000
Insertions	0	0	0
Deletions	0	0	0
Substitutions	227,913 (1.5%)	228,516 (1.5%)	227,279 (1.5%)

**Table 3:** Illumina long-clone reads statistics. Summary of the Illumina long-clone reads generated by MetaSim for each of the three synthetic metagenome datasets LC, MC and HC.

	LC-ilm-L	MC-ilm-L	HC-ilm-L
Simulated reads	200,000	200,000	200,000
Read length	75 bp	75 bp	75 bp
Clone length	1,900 bp	1,900 bp	1,900 bp
Simulated base pairs	15,000,000	15,000,000	15,000,000
Insertions	0	0	0
Deletions	0	0	0
Substitutions	227,880 (1.5%)	228,256 (1.5%)	228,262 (1.5%)

In total, we produced nine files of simulated reads:

• Roche-454 reads: LC-454, MC-454 and HC-454;

• Illumina reads, short clones: LC-ilm-S, MC-ilm-S and HC-ilm-S;

• Illumina reads, long clones: LC-ilm-L, MC-ilm-L and HC-ilm-L.

To be able to estimate the robustness of the results reported below, we additionally produced five replicates for each of the described datasets. Due to time constraints, in these replicates each Illumina datapoint was simulated using only 10,000 clones.

### MEGAN analysis of simulated reads

We performed a MEGAN analysis of all nine datasets. First, each of the datasets was compared against the NCBI-nr database (April 3, 2009 version) using BLASTX. Then, each of the nine BLASTX output files was parsed and analyzed using MEGAN, as described in more detail below.

### Analysis of Roche-454 reads

**Figure 2:**
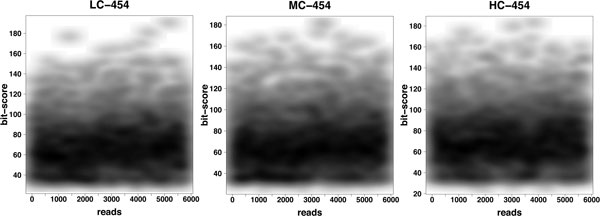
**Bit scores for Roche-454 datasets**. For each of the three simulated Roche-454 datasets, LC-454, MC-454 and HC-454, we plot the highest bit scores for all 6,000 reads.

The MEGAN analysis of the three different Roche-454 datasets, LC-454, MC-454 and HC-454, using the full NCBI-nr reference database, produced very few false negative species. Less than 6% of all species present in the synthetic metagenomes were not detected. Because of the low number of reads in each of the datasets (6,000 each), it is not surprising that some species of low abundance were missed. The false positive rate was zero for the LC-454 and HC-454 datasets, and less than 2% for the MC-454 dataset. Of course, the number of false negatives and the number of false positives both depend on the parameters applied, and the usual trade-off between false positives and false negatives can be observed. For these datasets, the best settings are *min score *= 50, *top percent *= 10 and the *min support *= 3. See Figure 2 for the distribution of bit scores for each of the three Roche-454 datasets.

**Figure 3:**
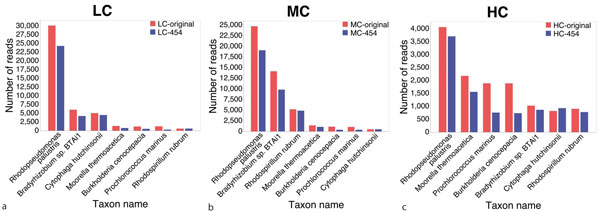
**Assignment of Roche-454 reads to taxa**. Blue bars indicate how many Roche-454 reads were assigned to seven different taxa, for each of the synthetic datasets LC (a), MC (b) and HC (c). Red bars indicate how many Roche-454 reads were actually simulated for each of the taxa. For ease of comparison, we have normalized the counts to a total of 100,000.

Note that this analysis addresses only the problem of detecting specific species in the dataset, not whether individual reads have been correctly assigned. To obtain an indication of how well the individual reads are assigned to the correct species, in Figure 3, we compare the number of reads assigned to specific species against the number of reads actually simulated for each species, for the seven most abundant species. We have normalized the data for this comparison. (The corresponding values for the five replicate datasets differ by between 10% (LC dataset) to 25% (HC dataset).)

#### Analysis of Illumina reads

All six files containing simulated Illumina reads, LC-ilm-S, MC-ilm-S, HC-ilm-S, LC-ilm-L, MC-ilm-L and HC-ilm-L, were compared against the NCBI-nr database using BLASTX and then analyzed using MEGAN's paired-read mode. In addition, to simulate single Illumina reads, we used the reads from our Illumina long clone files and processed them with MEGAN as single reads.

**Figure 4:**
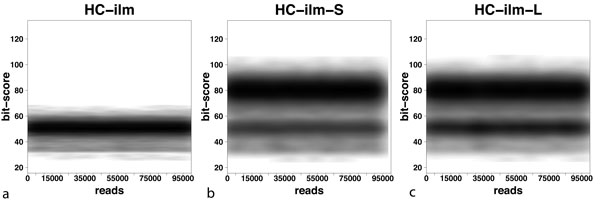
**Bit scores for Illumina reads**. For each of 100,000 (normalized) reads sampled from the HC synthetic metagenome, we plot the highest bit score attained for (a) Illumina single reads (HC-ilm), (b) Illumina short-clone pairs of reads (HC-ilm-S) and (c) Illumina long-clone pairs of reads (HC-lim-L). The latter two charts include the combined bit scores computed using equation (*). The plots for the LC and MC datasets look very similar and are therefore omitted.

In Figure 4, we show the distribution of bit scores for single Illumina reads on the synthetic HC dataset and compare it with the distribution of bit scores for both the short- and long-clone library. In the latter two charts, the dark bands centered at 80 bits are scores obtained by combining the scores of paired reads using equation (*), as implemented in MEGAN's paired-read mode. These plots clearly show the effect of combining matches from paired reads. The attained bit scores are much higher and it is clear that using a top percentage filter setting of 10% will make MEGAN use only those species that are hit by both reads of a pair in the LCA computation, when ever such hits are present. While the average combined bit scores are not as high as the bit scores reported for the simulated Roche-454 reads (see Figure 2), they are nevertheless much higher than the Illumina single read scores.

Consider a pair of reads *A *and *B *sequenced from the ends of the same clone. A main hypothesis of this paper is that more species will hit (that is, contain sequences that align to) both *A *and *B *if the reads come from a short clone than would be the case if the two reads come from a long clone. Our experimental study supports this claim. We observed that whenever the two reads of a short clone (≈ 200 bp) matched the same taxon, then this was due to matches to the same gene in over 80% of the cases, whereas for long clones (≈ 1,900 bp) this was true for just under 12%.

**Figure 5:**
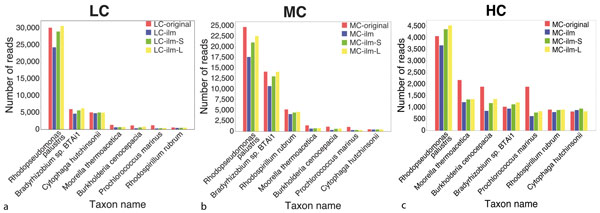
**Assignment of Illumina reads to taxa**. For seven key species, we indicate the number of simulated reads (red), along with the number of simulated Illumina single reads (blue), short-clone reads (green) and long-clone reads (yellow), assigned to the species by the LCA-gene content algorithm, for each of the three synthetic metagenome datasets LC (a), MC (b) and HC (c). (All values normalized to 100,000).

To obtain an indication of how well the individual reads are assigned to the correct species, Figure 5 we compare the number of reads assigned to specific species with the number of reads actually simulated for each species, for the same species as above. Here also we have normalized the data for comparison. (The corresponding values for the five replicate datasets differ by between 5% (LC dataset) to 25% (HC dataset).) In most cases, the number of assigned reads from long clones is larger than the number from short clones, which in turn is larger than the number of assigned single reads. In general, the number of false positive assignments is very small, except in the case of the HC dataset, where about 8% of the long-clone reads were falsely assigned to *Rhodopseudomonas palustris*.

**Figure 6:**
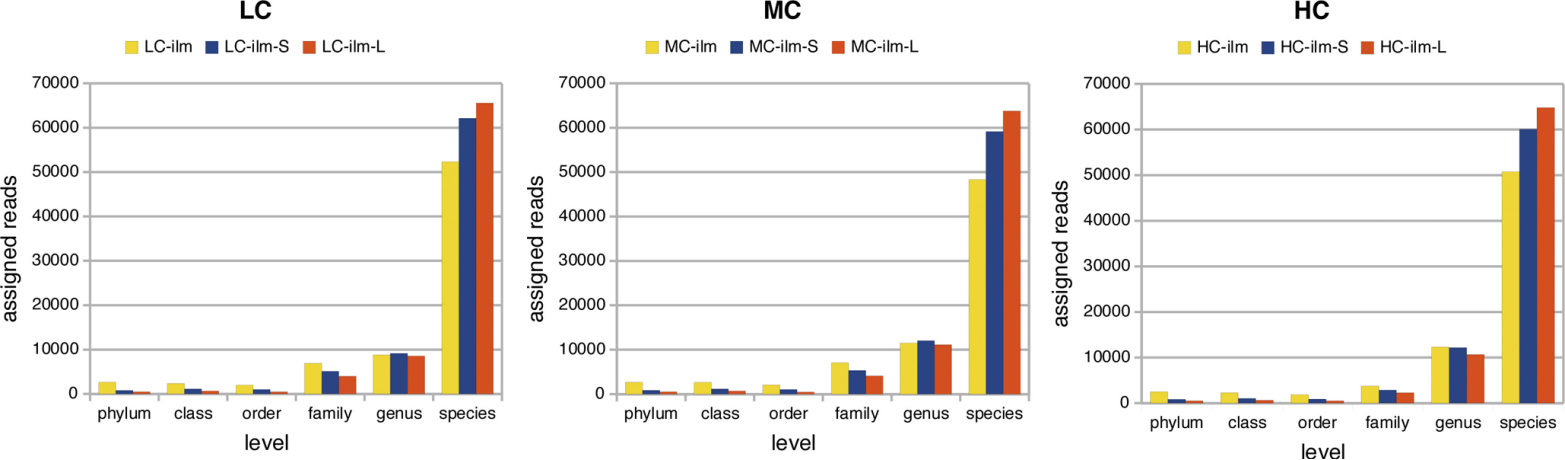
**Assignment of Illumina reads to taxonomic ranks**. The number of reads assigned to nodes at different ranks of the NCBI taxonomy, from the phylum level down to the species level. These numbers are reported for Illumina single reads (yellow), short-clone reads (blue) and long-clone reads (red), for each of the three synthetic metagenome datasets. All datasets normalized to 100,000 for ease of comparison.

Reads are assigned to nodes at different ranks of the NCBI taxonomy, depending on how conserved their sequence is across species. In Figure 6, we show the number of reads assigned to nodes at different ranks of the NCBI taxonomy, from the phylum level down to the species level.

The rate of false positive assignments to nodes of the different levels is very close to zero, and so we do not distinguish between correctly and falsely assigned reads in this figure. These charts indicate that the assignment of reads to taxa is most specific for Illumina long-clone reads, slightly less specific for short-clone reads and even less specific for single reads.

### The effect of unknown species

**Figure 7:**
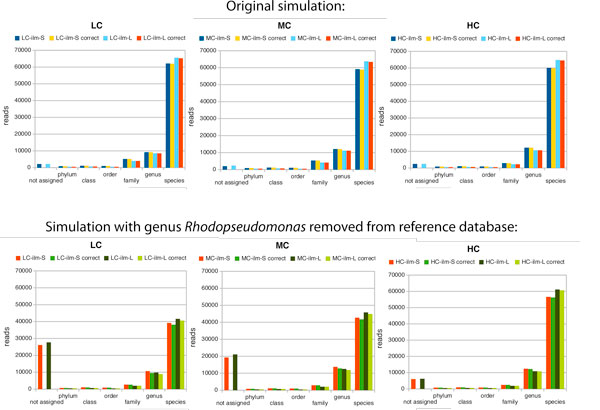
**Effect of removing a genus from reference database**. In the top row, we show the number of assigned and correctly assigned Illumina short- and long-clone reads at different taxonomical ranks. In the bottom row, we show the same quantities for a taxonomical analysis performed with the entire *Rhodopseudomonas *removed from the reference database.

The study described so far simulates the situation in which all organisms in the metagenome are represented by sequences in the reference database. In practice, a metagenome will usually contain a significant percentage of unknown organisms, which are not represented in the reference database. To mimic this situation, we decided to rerun the analysis while ignoring all BLAST matches to any taxon in the genus of *Rhodopseudomonas*. In Figure 7, we show the performance of MEGAN for both short clones and long clones. When using the whole of the NCBI-nr database as a reference, we can assign 60,000 - 65,000 reads at the species level, with a very small number of false positive assignments.

When we remove the genus of *Rhodopseudomonas *from the reference database, the percentage of reads assigned to species drops by a number roughly proportional to the number of reads that were actually sampled from the genus. In this case, the number of false positive assignments rises to about 2.5%, while most of the reads that were sourced from the "unknown" genus are classified as unassigned and are thus considered false negatives. This confirms that the LCA-gene content method for taxonomical analysis is indeed quite conservative in that unknown sequences are much more likely to produce false negatives than they are to produce false positives.

### Choice of MEGAN parameters

This taxonomical analysis of simulated Illumina reads was performed using the following MEGAN parameters: *min score *= 50, *top percent *= 10 and *min support *= 50. The most crucial parameter is the *min score*, which prescribes the minimal bit score that a match must achieve to be considered in the analysis. For single reads of short length, the program's recommended setting of this parameter is 35 bits. Figure 4 indicates that a *min score *of 40 or 45 might be more suitable, as it will be more specific, while still allowing most reads to be placed.

For paired-reads, Figure 4 suggests that using only those BLAST matches whose bit score exceeds 50 should perform very well. With this setting, for any pair of reads that has combined matches, only the combined matches will be used, as the bit scores of single-read matches will not pass the *top percent *filter. In cases where a pair of reads does not give rise to a pair of combined matches, then only very high-scoring single-read matches will be used.

**Figure 8:**
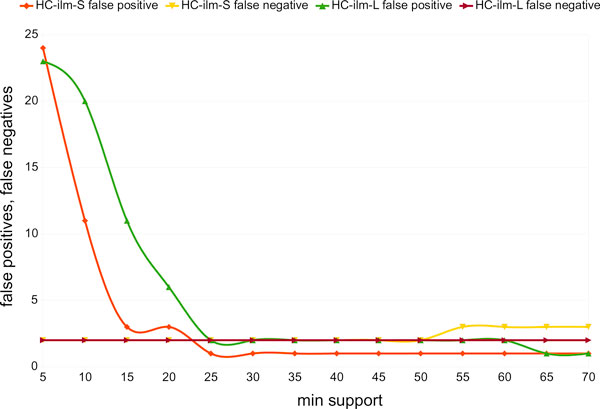
**Min-support settings**. For the synthetic HC metagenome, we report the number of false positives for the short clones (red) and long clones (green), and the number of false negatives for the short-clones (yellow) and the long clones (brown), as a function of the minimal number of hits required for a species to be considered detected.

To determine a recommended setting for the *min support *filter for Illumina paired reads, we studied the number of false positive and false negative assignments for both short-clones and long-clones for a number of different settings. All three synthetic metagenomes, LC, MC and HC, gave similar results, and so we only show the results for the HC dataset in Figure 8. Our studies suggest that *min support *= 50 is a good choice, as it minimizes both the number of false positives and false negatives.

### Comparison between Roche-454 and Illumina

**Figure 9:**
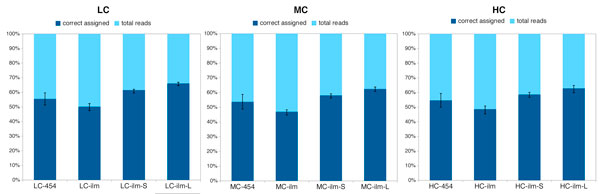
**Comparison of correctly assigned reads**. The percentage of correctly assigned reads (dark blue) to nodes at the species level of the NCBI taxonomy, averaged over the five replicate datasets, with error bars indicating the range of all five values. These numbers are reported for Roche-454 single reads (labeled 454), Illumina single reads (ilm), Illumina short-clone reads (ilm-S) and Illumina long-clone reads (ilm-L).

How do reads of length 250 bp compare against paired reads of length 75 bp? In Figure 9, for each of the three synthetic metagenomes, we report the number of reads that were correctly assigned on the species level, for Roche-454 sequencing, and Illumina single reads, short-clone reads and long-clone reads. In all cases, the number of falsely assigned reads is close to zero. Our study suggests that a higher percentage of Illumina paired reads than of Roche-454 single reads are correctly assigned to species.

As we argue above, long clones are more specific than short clones, because they lead to placements based on well-separated reads. This argument carries over to Roche-454 reads as well: While the reads are longer and thus support longer and more significant BLAST matches, the matches will usually reflect the gene content pattern of only one gene, rather than two.

How much of the difference between the results for the Roche-454 and the Illumina long-clones sequences is due to the different types of errors produced by the two different sequencing technologies? To investigate this, we generated an additional dataset covering all read lengths and clone lengths described above, but without applying any sequencing error models. For these error-free reads, an analysis analogous to Figure 9 exhibits a slightly different ranking of protocols by increasing performance, namely first short single reads, then short clones, then long single reads, then long clones. The gain of long-clone data (75 bp paired reads) over long single-read data (250 bp reads) is still significant at ≈ 4% (not shown).

## Competing interests

The authors declare that they have no competing interests.

## Authors' contributions

Suparna Mitra and Daniel Huson designed the project and wrote the manuscript. Max Schubach performed the simulation study and the analysis. All three authors participated in writing the necessary scripts and code.
